# Ameliorative Effects of Bovine Lactoferrin on Benzene-Induced Hematotoxicity in Albino Rats

**DOI:** 10.3389/fvets.2022.907580

**Published:** 2022-06-22

**Authors:** Mohamed F. Abou Elazab, Asmaa E. A. Elbaiomy, Mohamed S. Ahmed, Khalaf F. Alsharif, Naief Dahran, Ehab Kotb Elmahallawy, Abdallah A. Mokhbatly

**Affiliations:** ^1^Clinical Pathology Department, Faculty of Veterinary Medicine, Kafrelsheikh University, Kafr El-Sheikh, Egypt; ^2^Pathology Department, Faculty of Veterinary Medicine, Kafrelsheikh University, Kafrelsheikh, Egypt; ^3^Department of Clinical Laboratory Sciences, College of Applied Medical Sciences, Taif University, Taif, Saudi Arabia; ^4^Department of Anatomy, Faculty of Medicine, University of Jeddah, Jeddah, Saudi Arabia; ^5^Department of Zoonoses, Faculty of Veterinary Medicine, Sohag University, Sohag, Egypt

**Keywords:** bovine lactoferrin (bLf), benzene, hematotoxicity, albino rat, histopathological, hematological alterations

## Abstract

Benzene (Bz) is one of the major products of the petrochemical industry globally, which induces aplastic anemia and leukemia in humans and animals. This study aimed to investigate the modulatory effects of bovine lactoferrin (bLf) on Bz-induced hematotoxicity in albino rats. Eighty male rats were randomly divided into eight groups: corn oil group [2 mL/kg body weight (BW)], bLf groups (100, 200, and 300 mg/kg BW), Bz group (Bz 2 mL/kg BW; corn oil 2 mL/kg BW), and Bz + bLf groups (Bz 2 mL/kg BW; corn oil 2 mL/kg BW; bLf 100, 200, and 300 mg/kg BW). Hematobiochemical results exhibited marked pancytopenia, a significant decrease in total protein, albumin, α2- and γ-globulin, ferritin, serum iron, and total iron-binding capacity (TIBC), and an increase in serum bioactivities of aspartate aminotransferase, alanine aminotransferase, alkaline phosphatase (ALP), lactate dehydrogenase (LDH), and erythropoietin hormone levels in Bz-treated rats. Histopathological examination revealed a marked reduction in all hematopoietic cell lines in the bone marrow (BM), necrosis in the white pulp of the spleen and cytosolic hydrops, and apoptosis of hepatocytes in the Bz-treated group. Rats treated with bLf (300 mg/kg BW) revealed marked increases in total protein, albumin, α2- and γ-globulin, ferritin, serum iron, and TIBC levels and decreases both in ALP and LDH bioactivities and erythropoietin hormone levels compared with the Bz-treated group. Histopathological results were concomitant with hematobiochemical parameters in rats treated with bLf (300 mg/kg BW), almost showing restoration of the normal cellularity of BM, the architecture of red and white pulps of the spleen, and even the normal hypertrophy of hepatocytes compared with the control groups. To conclude, bLf (300 mg/kg BW) can be recommended to treat Bz-induced hematotoxicity.

## Introduction

Benzene (Bz), an aromatic hydrocarbon, is one of the natural constituents of crude oil and a product of petrol refining, which is emitted in large quantities from oil refineries (1, 2). Human exposure to Bz is through occupational and non-occupational sources. Occupational exposure is mostly reported in workers in leather, petrochemical (refining and service station operation), scientific laboratory, rubber, coal-based coke production, steel manufacturing, printing, and plastic manufacturing industries (3, 83). Nevertheless, cigarette smoke and ambient air are well-known sources of Bz nonoccupational exposure. Bz is an extensively used chemical in the petroleum industry. With its subsequent presence in the environment from other sources, human exposure to Bz is unavoidable, and possible adverse health effects associated with Bz's chronic or acute exposure remain a matter of great concern for the public (4).

Human exposure to Bz is associated with multiple adverse health effects, including alterations in hematological, hepatic, renal, lung, cardiac, immune system, nerve, and reproductive functions, with an increased risk of developing carcinogenesis (5, 6). Thus, Bz exposure can result in oxidative impairment, cell cycle arrest, apoptosis, and genotoxicity in the bone marrow [BM; (7, 8)]. Exposure to high Bz concentrations damages the BM, resulting in the decreasing number of erythrocytes, leukocytes, and/or thrombocytes in circulating blood, leading to aplastic anemia, as well as myelodysplasia, a preleukemic state, and leukemia (4, 9, 10). Bz exerts its hematotoxicity by the metabolic activation of toxic metabolites, such as 1,2-benzoquinone and hydroquinone, which affect the growth, gene expression, and apoptosis of hematopoietic cells (11, 12). Moreover, Bz hematotoxicity affects stromal cells, hindering their ability to produce normal cytokines to maintain normal hematopoietic cell growth and survival (11). Oxidative impairment by the dysregulated level of reactive oxygen species (ROS) can be the main mechanism, by which Bz exerts its toxicities (8). Bz metabolism can lead to the covalent binding of Bz metabolites to cellular macromolecules (DNA or protein) and the generation of oxygen stress by any of several mechanisms, including the effects of the formation of aromatic glutathionyl metabolites combined with interactions of specific metabolites, such as p-benzoquinone and muconaldehyde, thus, further suggesting that failure to repair the damage produced by any of these mechanisms may play some roles in generating BM impairment (13).

Bovine lactoferrin (bLf) is a natural iron-binding multifunctional glycoprotein expressed and secreted in large quantities in cow's milk. Numerous products containing bLf alone or in association with other nutraceuticals, supplements, or probiotics are currently being commercialized in human medicine (14). Oral administration of bLf is involved in numerous physiological and protective activities, including antioxidant (15), anti-inflammatory (16), anti-anemic (17), anticancer (18), immunomodulatory (19), and antimicrobial (20). Thus, bLf, through Fe^3+^ sequestration, controls the physiological balance of ROS production and their elimination rate and subsequently prevents the harmful effects of oxidative stress (14). To the best of the authors' knowledge, there is no specific and final drug to protect workers from occupational Bz-induced hematotoxicity. Given the above information, this study aimed to investigate the potential protective effects of bLf against Bz-induced hematotoxicity in albino rats and examine the effectiveness of bLf as a new therapeutic approach against Bz-induced hematotoxicity.

## Materials and Methods

### Chemicals

The Bz was purchased from Elgomhoryia Co. (Elmansora, Egypt). Bz was diluted with corn oil (1:1, v/v) before administration. The bLf was purchased from Wako Pure Chemical Industries (Osaka, Japan). The bLf was dissolved in distilled water (1 g/mL) and administered at 1 mL/kg body weight (BW) at different concentrations (100, 200, and 300 mg/kg BW) 30 min before Bz injection. All other chemicals are of analytical grade.

### Animals

Eighty male albino rats, each weighing 80–90 g, were obtained from the Animal Research Center (Mansoura, Egypt). Rats were kept in standard environmental conditions, housed in plastic cages, and acclimatized for 2 weeks in experimental room conditions at 25°C and a 12 h light/dark cycle. Rat diet and water were provided *ad libitum* throughout the experiment. All animal-related procedures were conducted according to the Ethical Committee of Kafrelsheikh University.

### Experimental Design

Rats were randomly divided into eight (four treated and four control) groups (10 rats each). Treated groups were as follows: Bz group [injected with Bz at 2 mL/kg (1,940 mg/kg) BW diluted v/v with corn oil, subcutaneous in the dorsal region], Bz + L100 group (received bLf orally at 100 mg/kg BW 30 min before Bz treatment), Bz + L200 group (received bLf orally at 200 mg/kg BW 30 min before Bz treatment), and Bz + L300 group (received bLf orally at 300 mg/kg BW 30 min before Bz treatment). Control groups were as follows: corn oil group (received the same volume of corn oil and distilled water as Bz and bLf vehicles subcutaneously and orally, respectively), L100 group (received bLf at 100 mg/kg BW), L200 group (received bLf at 200 mg/kg BW), and L300 group (received bLf at 300 mg/kg BW). All rats were treated 3 days weekly for seven consecutive weeks. All animals were sacrificed 24 h after the last Bz dose.

### Blood Sampling

Blood was collected from the retroorbital sinus of rats. Approximately 3 mL of blood were collected per animal, 1 mL was mixed immediately in tubes with Ethylenediaminetetraacetic acid EDTA for hematological analysis, and 2 mL was kept in plan tubes overnight at 4°C. Serum was separated from clotted blood *via* centrifugation at 4,000 × *g* for 15 min and stored at −20°C until use.

### Hematological Analysis

Using a Neubauer hemocytometer, red blood cell (RBC) and white blood cell counts were measured manually. Packed cell volume (PCV) or hematocrit was measured by capillary tubes using the micro-hematocrit method. Hemoglobin (Hb) concentration was determined by the cyano-methemoglobin method. Moreover, the mean corpuscular volume and mean corpuscular Hb were calculated mathematically using standard formulas (21). Differential leukocyte counts and platelet counts were determined microscopically from smears stained with a Wright–Giemsa stain.

### Biochemical Analysis

Serum aspartate aminotransferase (AST), alanine aminotransferase (ALT), alkaline phosphatase (ALP), and lactate dehydrogenase (LDH) were assayed using commercially available standard diagnostic kits (Spinreact, Santa Coloma, Spain). Total serum protein concentration was evaluated calorimetrically, and serum protein was fractionated by a semiautomated agarose gel electrophoresis system (Helena Laboratories, Helena Biosciences, UK) according to the manufacturer's instructions. Five protein fractions (albumins and α1-, α2-, β-, and γ-globulin) were identified and assessed in all serum samples, and absolute values for each fraction were mathematically obtained by multiplying the percentage by total protein concentration. Also, serum ferritin, iron, and total iron-binding capacity (TIBC) concentrations were measured using commercially available standard diagnostic kits (Spinreact). Erythropoietin levels were determined using a Rat EPO ELISA Kit (Elabscience, Houston, TX, USA) according to the manufacturer's instructions.

### BM Cellularity

After blood collection, animals were sacrificed by cervical dislocation, their femurs were removed quickly at necropsy and trimmed of muscular tissue; the femoral head and distal epiphysis were removed using a bone cutter. BM tissue was flushed five times with phosphate-buffered solution (PBS) (pH 7.2) containing 50% fetal bovine serum using a needle and syringe. BM smears (five smears per animal) were prepared immediately, and the slides were left to air-dry. The smear was stained by Giemsa stain (22).

### Histopathological Examination

All rats were sacrificed, a postmortem examination was performed, and all lesions were recorded. Specimens from all organs, especially the 1–2-cm core biopsy from the BM, liver, and spleen, were taken and kept in 10% neutral buffered formalin for histopathological examination. Specimens were dehydrated in ascending grades of alcohols, cleared in xylene, embedded in paraffin wax, sectioned at 4 μm, stained with hematoxylin and eosin stains, and examined *via* light microscopy (22, 23).

### Statistical Analysis

Data were statistically analyzed using a statistical software program (SPSS version 17.01 for Windows; SPSS, Inc., USA). Group data were compared using a one-way analysis of variance, followed by Duncan's test. Data of each study group were expressed as the mean ± standard deviation (SD), and *P* < 0.05 was considered statistically significant.

## Results

### Hematological Analysis

In Table 1, erythrogram data showed a significant decrease in the mean erythrocyte count, Hb concentration, and PCV percentage in the Bz-treated group compared with the control groups (*P* < 0.05), but there were no significant differences in the Bz + L-300 group compared with the control groups (*P* > 0.05). Also, leukogram data showed significant leukopenia, neutropenia, and lymphocytopenia in the Bz-treated group compared with the control groups (*P* < 0.05). However, the Bz + L300 group showed nonsignificant differences in total leukocyte and neutrophil counts compared with the control groups (*P* > 0.05). Moreover, significant lymphocytosis was observed in the Bz + L200 and Bz + L300 groups compared with the Bz-treated group (*P* < 0.05). Furthermore, neutrophil/lymphocyte ratios were significantly elevated in the Bz-treated group compared with the control groups (*P* < 0.05). However, the Bz + L300 group showed nonsignificant differences compared with the control groups (*P* > 0.05). Additionally, significant thrombocytopenia was detected in the Bz-treated group compared with the control groups (*P* < 0.05). Meanwhile, the Bz + L200 and Bz + L300 groups showed nonsignificant differences compared with the control groups (*P* > 0.05).

**Table 1:** Effect of bovine lactoferrin on hemogram in benzene-induced hematotoxicity.

**Groups**	**Parameters**								
	**RBC count (×10 ^**6**^/μ L)**	**Hb (gm/dL)**	**PCV (%)**	**MCV (fL)**	**MCH (pg)**	**WBC count (×10 ^**3**^/μ L)**	**Neutrophil Count (×10 ^**3**^/μ L)**	**Lymphocyte count (×10 ^**3**^/μ L)**	**Thrombocyte count (×10 ^**3**^/μ L)**
Corn oil	7.60 ± 0.24^a^	15.06 ± 0.34^a^	49.70 ± 1.11^a^	65.39 ± 1.88^a^	19.82 ± 0.57^a^	7.58 ± 0.49^ab^	1.85 ± 0.09^a^	5.04 ± 0.27^a^	962.40 ± 30.26^a^
L100	7.56 ± 0.29^a^	14.92 ± 0.44^a^	49.24 ± 1.46^a^	65.21 ± 2.44^a^	19.76 ± 0.74^a^	7.73 ± 0.66^ab^	1.91 ± 0.19^a^	5.07 ± 0.49^a^	964.20 ± 50.28^a^
L200	7.46 ± 0.36^a^	15.06 ± 0.27^a^	49.70 ± 0.89^a^	66.74 ± 4.01^a^	20.22 ± 1.22^a^	8.01 ± 0.46^a^	2.02 ± 0.15^a^	5.31 ± 0.49^a^	971.60 ± 46.06^a^
L300	7.54 ± 0.28^a^	15.22 ± 0.37^a^	50.23 ± 1.22^a^	66.65 ± 2.11^a^	20.19 ± 0.64^a^	8.07 ± 0.73^a^	2.05 ± 0.28^a^	5.48 ± 0.44^a^	975.80 ± 36.41^a^
Bz	4.92 ± 0.34^c^	9.46 ± 0.35^c^	30.79 ± 0.50^c^	62.84 ± 4.08^a^	19.27 ± 0.87^a^	3.40 ± 0.75^e^	1.23 ± 0.25^b^	1.78 ± 0.46^d^	561.40 ± 52.20^c^
Bz+L100	6.13 ± 0.98^b^	11.88 ± 1.54^b^	39.36 ± 5.22^b^	64.51 ± 2.70^a^	19.48 ± 0.89^a^	4.26 ± 0.91^d^	1.46 ± 0.25^b^	2.27 ± 0.48^d^	737.80 ± 104.37^b^
Bz+L200	6.38 ± 0.40^b^	12.42 ± 0.40^b^	40.99 ± 1.31^b^	64.49 ± 5.41^a^	19.54 ± 1.64^a^	5.92 ± 0.47^c^	1.77 ± 0.17^a^	3.38 ± 0.25^c^	912.20 ± 26.03^a^
Bze+L300	7.30 ± 0.41^a^	14.40 ± 0.76^a^	47.73 ± 2.53^a^	65.46 ± 2.36^a^	19.75 ± 0.73^a^	6.97 ± 0.52^b^	1.89 ± 0.09^a^	4.45 ± 0.33^b^	939.80 ± 49.11^a^

*Corn group, received corn oil and distilled water as the Bz and bLf vehicles; L100 group, received bLf (100 mg/kg BW); L200 group, received bLf (200 mg/kg BW); L300 group, received bLf (300 mg/kg BW); Bz group, injected with Bz at a dosage of 2 ml/kg BW; Bz+L100 group, received bLf (100 mg/ kg BW, 30 min before Bz treatment); Bz+L200 group, received bLf (200 mg/ kg BW, 30 min before Bz treatment), and Bz+L300 group, received bLf (300 mg/ kg BW, 30 min before Bz treatment). Data are expressed as means ± SD. Mean values in the same row bearing different superscript letters were significantly differ at (P < 0.05)*.

### Biochemical Analysis

In Table 2, serum bioactivities of AST, ALT, ALP, and LDH significantly increased in the Bz-treated group compared with the control groups (*P* < 0.05). However, the Bz + L-300 group showed a significant decrease compared with the Bz-treated group (*P* < 0.05). Also, total protein, albumin, and α2- and γ-globulin concentrations significantly decreased in the Bz-treated group compared with the control groups (*P* < 0.05). However, the Bz + L200 and Bz + L300 groups showed nonsignificant differences compared with the control groups (*P* > 0.05). Moreover, serum ferritin, iron, and TIBC concentrations significantly decreased in the Bz-treated group compared with the control groups (*P* < 0.05), as shown in Table 3. However, serum erythropoietin concentrations significantly increased in the Bz-treated group compared with the control groups (*P* < 0.05). Moreover, serum ferritin and TIBC concentrations in the Bz + L200 and Bz + L300 groups significantly increased compared with the Bz-treated group (*P* < 0.05). Also, serum erythropoietin concentrations significantly decreased in the Bz + L200 and Bz + L300 groups compared with the Bz-treated group (*P* < 0.05).

**Table 2:** Effect of bovine lactoferrin on enzymes bioactivities and serum protein electrophoresis in benzene-induced hematotoxicity.

**Groups**	**Parameters**									
	**AST (U/L)**	**ALT (U/L)**	**ALP (U/L)**	**LDH (U/L)**	**Total protein (gm/dl)**	**Albumin (gm/dl)**	**α1-globulin (gm/dl)**	**α2- globulin (gm/dl)**	**β- globulin (gm/dl)**	**γ- globulin (gm/dl)**
Corn oil	205.67 ± 9.29^c^	121.33 ± 11.02^e^	238.67 ± 22.59^d^	2407.67 ± 97.21^bc^	7.30 ± 0.46^bc^	5.08 ± 0.45^bc^	0.16 ± 0.01^abc^	0.54 ± 0.04^ab^	0.58 ± 0.04^ab^	0.94 ± 0.04^b^
L100	214.67 ± 14.15^c^	115.00 ± 10.44^e^	233.67 ± 25.66^d^	2377.33 ± 65.58^bc^	7.99 ± 0.17^a^	5.66 ± 0.08^a^	0.17 ± 0.03^bc^	0.62 ± 0.04^a^	0.60 ± 0.04^ab^	0.96 ± 0.08^b^
L200	195.00 ± 21.66^cd^	127.00 ± 1.00^e^	224.67 ± 50.66^d^	2416.33 ± 250.51^bc^	7.83 ± 0.32^ab^	5.46 ± 0.24^ab^	0.16 ± 0.03^abc^	0.58 ± 0.02^ab^	0.63 ± 0.03^a^	1.04 ± 0.09^ab^
L300	180.33 ± 15.82^d^	114.67 ± 11.50^e^	222.67 ± 32.04^d^	2204.00 ± 143.27^c^	7.81 ± 0.29^abc^	5.43 ± 0.14^abc^	0.14 ± 0.03^abc^	0.57 ± 0.05^ab^	0.57 ± 0.04^ab^	1.08 ± 0.05^a^
Bz	328.33 ± 1.53^a^	277.33 ± 8.09^a^	457.00 ± 31.43^a^	2797.67 ± 155.05^a^	5.83 ± 0.35^e^	3.91 ± 0.26^e^	0.12 ± 0.01^c^	0.44 ± 0.04^c^	0.55 ± 0.03^b^	0.81 ± 0.06^c^
Bz+L100	310.00 ± 3.00^a^	195.33 ± 5.51^b^	396.67 ± 21.39^b^	2588.67 ± 54.20^ab^	6.59 ± 0.19^d^	4.47 ± 0.14^d^	0.13 ± 0.00^b^	0.51 ± 0.02^b^	0.57 ± 0.04^ab^	0.90 ± 0.02^bc^
Bz+L200	281.33 ± 3.21^b^	175.00 ± 11.14^c^	380.00 ± 10.54^bc^	2374.67 ± 219.14^bc^	7.14 ± 0.37^cd^	4.95 ± 0.27^c^	0.14 ± 0.01^ab^	0.52 ± 0.04^b^	0.61 ± 0.03^ab^	0.93 ± 0.04^bc^
Bze+L300	269.67 ± 1.53^b^	147.67 ± 12.50^d^	340.33 ± 26.65^c^	2346.00 ± 46.78^bc^	7.50 ± 0.56^abc^	5.08 ± 0.38^bc^	0.15 ± 0.01^abc^	0.57 ± 0.05^ab^	0.60 ± 0.06^ab^	1.00 ± 0.10^ab^

*Corn group, received corn oil and distilled water as the Bz and bLf vehicles; L100 group, received bLf (100 mg/kg BW); L200 group, received bLf (200 mg/kg BW); L300 group, received bLf (300 mg/kg BW); Bz group, injected with Bz at a dosage of 2 ml/kg BW; Bz+L100 group, received bLf (100 mg/ kg BW, 30 min before Bz treatment); Bz+L200 group, received bLf (200 mg/ kg BW, 30 min before Bz treatment), and Bz+L300 group, received bLf (300 mg/ kg BW, 30 min before Bz treatment). Data are expressed as means ± SD. Mean values in the same row bearing different superscript letters were significantly differ at (P < 0.05)*.

**Table 3:** Effect of bovine lactoferrin on erythropoietin and serum iron profile in benzene-induced hematotoxicity.

**Groups**	**Parameters**			
	**Erythropoietin ng/L**	**Serum ferritin pmol/L**	**Serum iron μmol/L**	**TIBC μmol/L**
Corn oil	3.97 ± 0.15^c^	26.23 ± 1.57^d^	170.33 ± 2.52^c^	357.67 ± 5.13^c^
L100	4.36 ± 0.31^c^	34.53 ± 2.35^c^	180.33 ± 2.31^b^	367.67 ± 2.52^bc^
L200	4.50 ± 0.17^c^	40.17 ± 2.20^b^	192.67 ± 5.51^a^	378.33 ± 5.03^ab^
L300	4.93 ± 0.38^c^	52.40 ± 3.70^a^	196.00 ± 7.21^a^	389.33 ± 5.03^a^
Bz	11.18 ± 0.98^a^	10.73 ± 1.23^f^	92.67 ± 5.51^f^	281.67 ± 7.77^e^
Bz+L100	10.71 ± 0.79^a^	16.10 ± 1.64^e^	119.67 ± 4.51^e^	310.00 ± 13.23^d^
Bz+L200	8.55 ± 0.48^b^	29.22 ± 3.40^d^	134.00 ± 4.58^d^	355.00 ± 8.66^c^
Bze+L300	8.10 ± 0.35^b^	43.03 ± 2.64^b^	176.00 ± 6.56^bc^	388.67 ± 2.52^a^

*Corn group, received corn oil and distilled water as the Bz and bLf vehicles; L100 group, received bLf (100 mg/kg BW); L200 group, received bLf (200 mg/kg BW); L300 group, received bLf (300 mg/kg BW); Bz group, injected with Bz at a dosage of 2 ml/kg BW; Bz+L100 group, received bLf (100 mg/ kg BW, 30 min before Bz treatment); Bz+L200 group, received bLf (200 mg/ kg BW, 30 min before Bz treatment), and Bz+L300 group, received bLf (300 mg/ kg BW, 30 min before Bz treatment). Data are expressed as means ± SD. Mean values in the same row bearing different superscript letters were significantly differ at (P < 0.05)*.

### BM Cellularity

In Table 4, nucleated cells in the BM were significantly lower in the Bz-treated group than in the control groups (*P* < 0.05). Moreover, the Bz + L100 group showed a nonsignificant increase in BM cellularity than the Bz-treated group (*P* > 0.05; Table 4). However, nucleated cells in the BM of the Bz + L200 and Bz + L300 groups showed nonsignificant differences compared with the control groups (*P* > 0.05; Table 4).

**Table 4:** Effect of bovine lactoferrin on bone marrow cellularity in benzene-induced hematotoxicity.

**Groups**	**Parameters**
	**Nucleated cell %**
Corn oil	8.45 ± 1.82^a^
L100	8.40 ± 1.62^a^
L200	8.49 ± 1.46^a^
L300	8.57 ± 1.38^a^
Bz	5.14 ± 1.17^c^
Bz+L100	6.50 ± 1.58^b^
Bz+L200	7.12 ± 1.62^b^
Bze+L300	8.55 ± 1.50^a^

*Corn group, received corn oil and distilled water as the Bz and bLf vehicles; L100 group, received bLf (100 mg/kg BW); L200 group, received bLf (200 mg/kg BW); L300 group, received bLf (300 mg/kg BW); Bz group, injected with Bz at a dosage of 2 ml/kg BW; Bz+L100 group, received bLf (100 mg/ kg BW, 30 min before Bz treatment); Bz+L200 group, received bLf (200 mg/ kg BW, 30 min before Bz treatment), and Bz+L300 group, received bLf (300 mg/ kg BW, 30 min before Bz treatment). Data are expressed as means ± SD. Mean values in the same row bearing different superscript letters were significantly differ at (P < 0.05)*.

### Histopathological Examination

**Figure 1:**
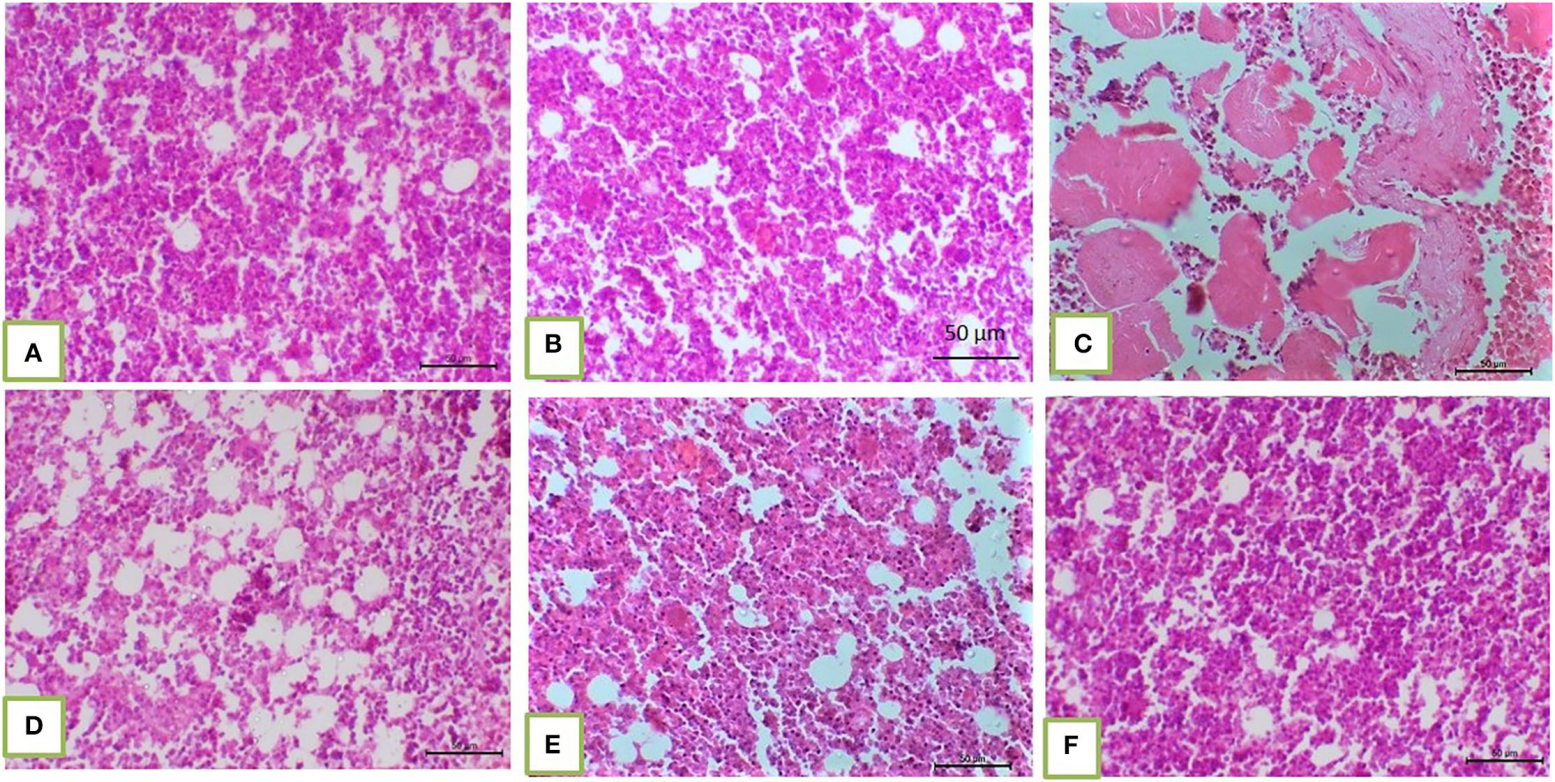
BM biopsy demonstrated normal cellular BM separated from each other by fat vacuoles in corn oil-treated groups and those treated with 100, 200, and 300 mg/kg BW bLf [**(A)** and **(B)**, respectively]. Hypocellular BM with residual islands of hematopoietic cells in the Bz-treated group **(C)**. Slight increase in BM cellularity in the Bz + 100 mg/kg BW bLf group **(D)**. More cellular BM in Bz + 200 mg/kg BW bLf **(E)**. The cellular density of BM became almost similar to the control groups **(F)**.

**Figure 2:**
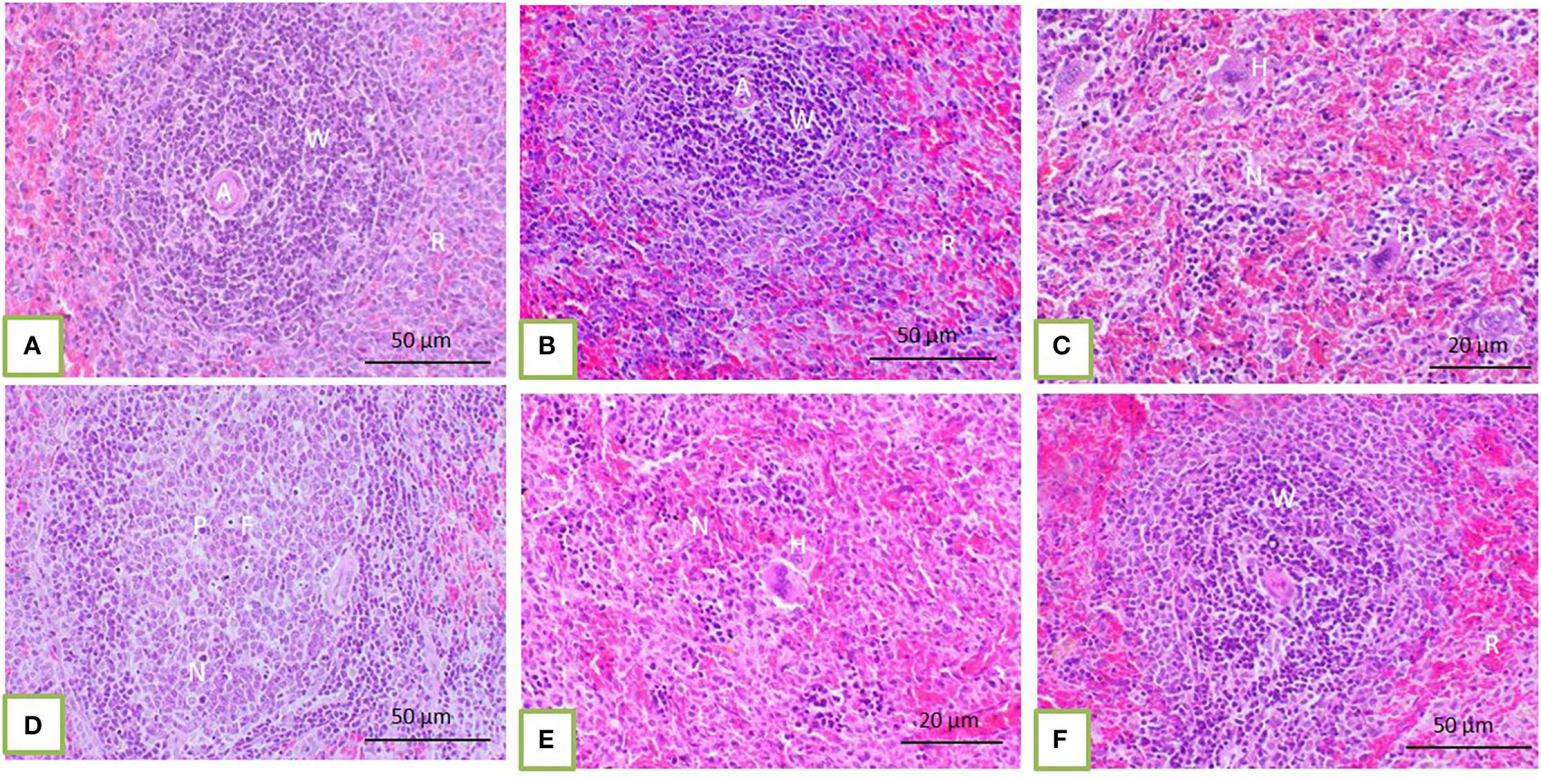
The spleen of control rats and those treated with 100, 200, and 300 mg/kg BW bLf showed distinct white and red pulps. The white pulp (W) comprises small, medium, and large lymphocytes and plasma cells with intact arteriole **(A)**. The red pulp (R) is composed of venous sinuses with various cell types **(A,B)**. The spleen of rats treated with Bz showed a moderate degree of EMH (H) in the red pulp **(C)** in addition to apoptotic hematopoietic cells (P) and necrotic areas (N) containing foamy macrophages **(F)** present among white and red pulps **(D)**. The spleen of rats treated with Bz + 100 and 200 mg/kg BW bLf showed less EMH **(E)**. However, the spleen of rats treated with Bz + 300 mg/kg BW bLf showed similar morphology to that in the control group **(F)**.

**Figure 3:**
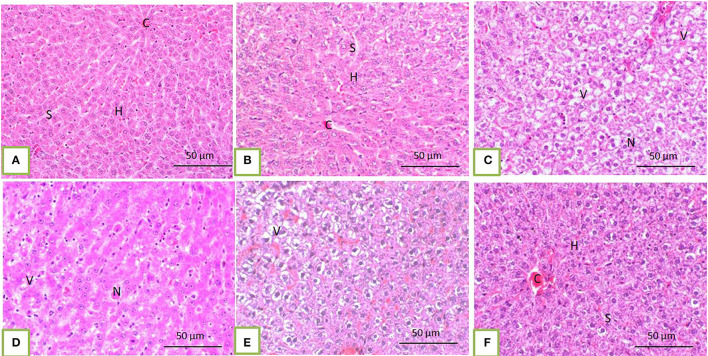
The liver of control rats and those treated with 100, 200, and 300 mg/kg BW bLf showed a normal pattern of hepatic lobule with a centrally located vein **(C)** and arrangement of hepatocytes (H) in cords separated from each other by hepatic sinusoids [S; **(A)** and **(B)**]. In rats treated with Bz alone, the liver showed marked vacuolation of hepatocytes (V) and focal hepatocytic necrosis [N; **(C)** and **(D)**]. In rats treated with Bz + 100 and 200 mg/kg BW bLf, the liver showed slight cytoplasmic vacuolation **(E)**. However, in rats treated with Bz + 300 mg/kg BW bLf, the liver showed the same morphology as the control group **(F)**.

Histopathological core biopsy examination of the BM of rats treated with corn oil and those with 100, 200, and 300 mg/kg BW bLf showed the same picture of normal cellularity of BM (Figures 1A,B). Rats treated with Bz showed a marked reduction in all hematopoietic cell lines in the BM (Figure 1C). Rats treated with Bz + 100 and 200 mg/kg BW bLf showed similar results, with small, scattered islands of hematopoietic cells (Figures 1D,E). Meanwhile, rats treated with Bz and bLf 300 showed cellular BM almost similar to the control ones with more cellular density BM (Figure 1F). The spleen of rats treated with corn oil and those treated with 100, 200, and 300 mg/kg BW bLf showed the same picture of a normal spleen (Figures 2A,B). Rats treated with Bz alone showed the presence of extramedullary hematopoietic (EMH) cells containing irregular nests and patches of nucleated hematopoietic cells of erythroid and myeloid cells and numerous megakaryocytes (Figure 2C). Apoptotic hematopoietic cells and necrotic areas with foamy macrophages were noticed among white pulp cells in rats treated with Bz (Figure 2D). There was less EMH and apoptotic cells in both groups treated with Bz + 100 and 200 mg/kg BW bLf (Figure 2E). However, rats treated with Bz and bLf 300 showed the normal architecture of red and white pulps (Figure 2F). By contrast, the liver of control rats and those treated with 100, 200, and 300 mg/kg BW bLf showed normal lobular organization with hepatocytes arranged in hepatic cords separated by sinusoids (Figures 3A,B). However, the liver of rats treated with Bz showed cytosolic hydrops of cells in peripheral areas of hepatic lobules (Figure 3C) and many apoptotic hepatocytes with condensed eosinophilic cytoplasm and pyknotic nuclei (Figure 3D). Rats treated with Bz and bLf 100 and 200 showed less cytosolic hydrops in the hepatocytes (Figure 3E). However, rats treated with Bz and 300 mg/kg BW bLf showed normal even hypertrophy of hepatocytes with diploid nuclei (Figure 3F).

## Discussion

Chronic Bz exposure leads to failures in the BM environment, reducing the circulating counts of erythrocytes, leukocytes, platelets, pancytopenia, aplastic anemia, myelodysplasia, and myelogenous leukemia (10). By contrast, there is no effective therapeutic approach to protect industrial workers and other individuals from Bz-induced hematotoxicity. Experimental aplastic anemia models in different species, doses, routes of administration, and exposure times should be induced to study the disease at different stages under controlled conditions and examine curative therapeutic alternatives (24, 25). In mammals, Bz toxicity depends mainly on metabolism, which takes place in the liver, where it is primarily metabolized into Bz oxide by cytochrome P450, which is bio-converted to muconaldehyde and phenol. Phenol metabolites undergo further metabolism to form catechol and hydroquinone; the latter in the BM is converted into benzoquinones due to its high myeloperoxidase level, leading to damage in BM hematopoietic stem cells and resulting in aplastic anemia. At the same time, the BM-related myeloperoxidase produces some quinones and semiquinones, which, besides directly binding to macromolecules, can produce oxygen free radicals by the redox reaction (26–29).

In this study, rats were subcutaneously injected with Bz at 2 mL/kg thrice weekly for seven consecutive weeks to demonstrate Bz-induced hematotoxicity. This study also investigated the protective effects of bLf in reducing Bz-induced hematotoxicity in the BM and peripheral blood cells. There was a decrease in the number of all hematopoietic cell lines in the BM of rats treated with Bz, in agreement with previous studies (30, 31) that some chemotherapeutics, antibiotics, antihypertensives and phenylbutazone, phenobarbital, and griseofulvin can induce myelopoietic cell decreases to occur less frequently than erythroid-related hypoplasia. Besides the decrease in BM cellularity, there was a decrease in peripheral blood cells characterized by anemia, leukopenia, neutropenia, lymphocytopenia, and thrombocytopenia, consistent with previous studies in a mouse model (32–36).

The most important noticeable histopathological changes in the spleen were the presence of EMH cells and numerous apoptotic hematopoietic cells in the spleen of rats treated with Bz. The degree of EMH and apoptotic cells decreased in groups treated with Bz + 100 and 200 mg/kg BW bLf. However, rats treated with Bz + 300 mg/kg BW bLf restored the normal architecture of red and white pulps. Exposure of BM to severe stress with Bz may have resulted in releasing platelet stem cells into the circulation, reaching the spleen for subsequent proliferation and differentiation (37). Also, Bz affected the spleen structure and organization, leading to the accumulation of megakaryocytes, as the spleen became unable to function as an alternate hematopoietic organ with platelets, liberation was impaired, and megakaryocytes accumulated in the spleen (38). Previous studies (39, 40) mentioned that hematopoiesis occurs outside the BM, including the spleen and liver, as a result of pathophysiological alterations in hematopoietic stem/progenitor cells, as well as the ectopic emergence of their niche in these tissues, a process called EMH. The observed necrotic areas in a white pulp with foamy macrophages might indicate spleen toxicity, which probably impaired the efficient erythrocyte removal (41). These macrophages might contain remnants of phagocytized deformed RBCs and platelets, as the spleen normally regulates peripheral erythrocyte and platelet counts by removing aged or damaged cells. Bz exposure may have impaired this function, causing the accumulation of circulating platelets despite severe BM hypoplasia and impaired platelet and megakaryocyte production (42).

The liver is the primary organ concerned with the biotransformation of chemicals, which mostly results in markedly detrimental effects on the biochemistry of hepatocytes, such as excessive ROS generation, which causes oxidative damage not only in the liver but also in other organs, including the kidneys, spleen, and hematopoietic system (36, 43–50). In this study, Bz-treated rats revealed significant increases in serum bioactivities of AST, ALT, ALP, and LDH and decreased serum total protein, albumin, and α2- and γ-globulin concentrations. Transaminases and phosphatases are important critical enzymes in biological processes and are considered specific biochemical indicators of liver damage (51, 52). AST, ALT, and LDH are important to the metabolism of cellular nitrogen, liver glucose, and amino acid oxidation. They are found in hepatocytes, the heart, kidney, skeletal muscle, and pancreas. Increasing the activity of these enzymes plays an important role in amino acid oxidation or transformation during gluconeogenesis (53). ALP plays an integral role in glycogen metabolism in the liver through stimulation of glucose synthesis to overcome the energy required during stress conditions. Therefore, ALP has been used as an indicator of liver damage and a hallmark for liver dysfunction (13, 52, 54). Elevation of ALT activity appears to reflect hepatic diseases more specifically than AST values. The activity of either enzyme, particularly AST, may also be elevated in extrahepatic diseases. However, the elevation of AST and ALT, along with the elevation of ALP activities, may reflect some necroinflammatory diseases of the liver (55). Elevation of liver enzymes might result from hepatocyte membrane damage due to increased ROS levels and the glutathione depletion effects of Bz metabolites (Bz oxide and hydroquinone) in hepatocytes, resulting in disturbances in the function of enzymes and other elements of cells. Several enzymes located in the hepatocyte cytosol are released into the bloodstream. This is positively correlated with observed histopathological changes in the liver tissue and agrees with the findings described elsewhere (56–58). The noticed histopathological changes in the liver of rats treated with Bz in the form of cytosolic vacuolation and sinusoidal congestion were in agreement with similar reports of exposure to Bz or its derivatives in mice (59), rats (60), and workers exposed to Bz (61).

This study also showed a significant decrease in serum total protein, albumin, and α2- and γ-globulin concentrations in the Bz-treated group. The laboratory estimation of serum protein concentrations is a vital component of laboratory diagnostic evaluations in different animals. Protein alterations commonly occur as secondary changes in various diseases. Abnormal protein concentrations may result from alterations in the albumin or globulin concentrations (or both). In hepatocellular damage, albumin concentrations may be decreased concurrently due to decreased hepatic synthesis (62). However, decreased albumin may be attributed to several factors, including malnourishment, gastrointestinal diseases, intestinal parasitism, increased catabolism, and renal diseases (63, 64). Additionally, albumin concentration falls gradually during infectious and inflammatory diseases (65). Furthermore, a decrease in γ-globulin concentrations may represent the suppression of immunoglobulin- or antibody-producing cells (B lymphocytes) by Bz. Immune deficiency involving B lymphocytes or plasma cells can result in low immunoglobulin concentrations and, in some cases, hypoglobulinemia (62).

Furthermore, data showed a significant increase in erythropoietin levels in the Bz-treated group. Erythropoietin, the principal hormonal regulator of erythropoiesis, is required to develop later stages of erythroid precursors and the production of mature RBCs (66). Increases in severe hypoxia result indirectly from lowered Hb concentrations, up to 1,000-fold (67). By contrast, its concentration significantly decreased in the Bz + L200 and Bz + L300 groups compared with the Bz-treated group. In this study, the serum iron profile in Bz-induced hematotoxicity showed a significant decrease in serum ferritin, iron, and TIBC. Hypoferremia in serum iron is related to absorptive failures, nutritional deficiencies, iron loss *via* chronic external blood loss, hypoproteinemia, and aberrant iron metabolism with a shift to storage sites (macrophages) at the expense of hematopoietic cells during chronic disease processes and inflammation (68). Decreased serum TIBC usually occurs in inflammation and hepatic insufficiency. Apotransferrin is a negative acute-phase protein (its production is decreased by the action of inflammatory mediators); therefore, inflammation may lead to hypotransferrinemia. Also, because transferrin is a β-globulin produced by hepatocytes, a liver disease that causes hypoproteinemia may cause hypotransferrinemia (69).

The protective effects of bLf against Bz-induced hematotoxicity were studied by administering bLf at three dose levels (100, 200, and 300 mg/kg BW) simultaneously with Bz. Results showed an improvement in hematological parameters and BM cellular density, which were slightly restored with a gradual increase in the dose of bLf and were almost similar to the values in control rats treated with 300 mg/kg BW bLf, in agreement with a previous study (70). The protective effects of bLf against Bz-induced hematotoxicity could be due to its antioxidative, anti-inflammatory, immunomodulatory, and antiapoptotic properties. Lactoferrin, a nonheme iron-binding protein of the transferrin family with a high affinity for iron, inhibits ROS overproduction due to its iron-binding capacity, as iron promotes hydroxyl radical formation and lipid oxidation during inflammation (71–73). The anti-inflammatory effect of bLf is mainly attributed to its significant role in the induction of anti-inflammatory cytokines interleukin (IL)-4 and IL-10, and reductions in the proinflammatory cytokines tumor's necrosis factor-α and IL-1β by suppressing NF-κB signaling pathway (74). Also, bLF has been reported to promote the macrophage shift from inflammatory to tolerogenic phenotype, which is key for tissue homeostasis (75). In addition, lactoferrin can reduce oxidative stress-induced apoptosis by declining the intracellular levels of ROS (76). Moreover, Lf antioxidant activity is most likely related to its iron scavenging ability and inhibition of iron-catalyzed formation of ROS (72, 73). Furthermore, Lf enhances the proliferation, differentiation, maturation, migration, and function of immune cells (77). In this study, hepatic tissue became healthy in groups treated with BZ + 300 mg/kg BW bLf, which might be attributed to the antioxidant effects of bLf. This was documented in the prevention of Aβ-induced oxidative stress and Alzheimer's disease (78), which helps in metabolic adaptation against the toxic metabolite, mainly the ROS produced by Bz, and subsequent regenerative cell proliferation of hepatocytes (79–81). Also, antioxidant enzyme levels depend on the availability of antioxidants in the diet, and to achieve the proper function of these enzymes, adequacy of some micronutrients, such as bLf, is essential (76, 81, 82).

## Conclusions

This study showed protective effects of bLf against Bz-induced hematotoxicity in albino rats through the amelioration of hematological disorders and reduced histological damages in the spleen and liver. This study recommended bLf as a protective supplement to reduce Bz-induced hematotoxicity.

## Data Availability Statement

The original contributions presented in the study are included in the article/supplementary material, further inquiries can be directed to the corresponding authors.

## Ethics Statement

This study was conducted with the approval of the Faculty of Veterinary Medicine, Kafrelsheikh University and the Institutional Review Board Number KFS-2021/03..

## Author Contributions

ME, AE, MA, and AM designed the idea of the conception, performed the methodology, formal analysis, data curation, and contributed their scientific advice and supervision besides revision of the manuscript. KA, ND, and EE drafted the manuscript, contributed their scientific advice, and prepared the manuscript for publication and revision. The manuscript has been read and approved by all the authors.

## Funding

This work was supported by the Taif University Researchers Supporting Program (Project Number: TURSP-2020/153), Taif University, Saudi Arabia.

## Conflict of Interest

The authors declare that the research was conducted in the absence of any commercial or financial relationships that could be construed as a potential conflict of interest.

## Publisher's Note

All claims expressed in this article are solely those of the authors and do not necessarily represent those of their affiliated organizations, or those of the publisher, the editors and the reviewers. Any product that may be evaluated in this article, or claim that may be made by its manufacturer, is not guaranteed or endorsed by the publisher.
